# External Validation of the NECPAL CCOMS-ICO Prognostic Tool for Early Palliative Care and Mortality Prediction in Patients with Advanced Chronic Conditions: A Prospective Observational Study Protocol

**DOI:** 10.3390/mps8030045

**Published:** 2025-05-01

**Authors:** Ana Bustamante-Fermosel, Elena Díaz-Sánchez, Natalia Pavón-Muñoz, Laetitia Hennekinne, Fuensanta Gil-Gil, Helena Notario-Leo, Alicia Sánchez-Pizarro, Marta Bustamante-Vega, Juan Torres-Macho, Anabel Franco-Moreno, on behalf of the COMPASS Research Group

**Affiliations:** 1 Faculty of Medicine, Universidad Complutense de Madrid, 28233 Madrid, Spain; 2 Internal Medicine Department, Hospital Universitario Infanta Leonor-Virgen de la Torre, 28031 Madrid, Spain; 3 Palliative Care, Hospital Universitario Infanta Leonor-Virgen de la Torre, 28031 Madrid, Spain; 4 Nursing Unit, Hospital Universitario Infanta Leonor-Virgen de la Torre, 28031 Madrid, Spain; 5 Department of Internal Medicine, Hospital Universitario Infanta Leonor-Virgen de la Torre, 28031 Madrid, Spain

**Keywords:** advanced chronic illnesses, external validation, quality of life, mortality, NECPAL prognostic tool, palliative care needs, patient care planning, prognosis

## Abstract

Early initiation of palliative care in patients with advanced chronic conditions significantly improves their quality of care; however, variability in disease trajectories complicates such interventions’ timing. The NECPAL CCOMS-ICO prognostic tool was developed as a straightforward instrument to help healthcare providers in all clinical settings promptly identify patients with advanced chronic conditions who require palliative care, thereby enhancing service planning and delivery. Its latest version, 4.0, 2021, for the first time, incorporates a patient survival estimation. Nevertheless, validation is necessary. This study aims to validate the NECPAL version 4.0 tool in an independent cohort. It is an observational, prospective study involving outpatients and hospitalized non-randomized patients at Hospital Universitario Infanta Leonor–Virgen de la Torre in Madrid, Spain, all of whom have at least one advanced chronic condition. The study is scheduled to last 6 years, including a recruitment period of 30 months starting 1 February 2024, followed by a 12-month follow-up period for each patient. This is the first prospective study designed to validate the NECPAL version 4.0 instrument. Implementing this tool would allow the identification of patients with advanced chronic conditions and unmet palliative care needs and determine the more appropriate care pathway at the proper moment.

## 1. Introduction

In economically developed countries, most people now die from complex long-term illnesses such as cancer, respiratory and heart diseases, stroke, and dementia [1]. These conditions are characterized by gradual progression, prolonged duration, and prognostic uncertainty. Identification of patients with a life-limiting illness when they are starting to need a change in their goals of care contributes to end-of-life care planning.

**Figure 1:**
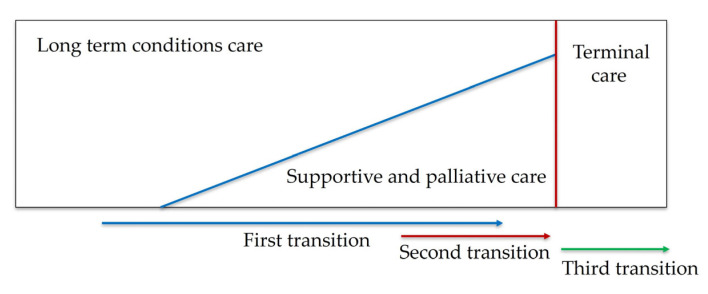
Early integration model of palliative care.

The World Health Organization (WHO) underlines the importance of providing palliative care (PC) early in the disease trajectory of patients suffering from a life-limiting illness [2]. Evidence shows earlier access to PC promotes quality of life, reduces hospitalizations, and even prolongs survival [3,4,5,6,7,8,9,10,11]. However, the WHO estimates that only 14% of patients with life-limiting diseases who need PC receive it [4]. In this setting, patients should not only be treated at the final stages of their disease but before. The concepts of “first transition”, “second transition”, and “third transition” have been developed to address the evolving needs of patients with advanced chronic illnesses (Figure 1). The “first transition” emphasizes integrating PC at the onset of a chronic or life-limiting diagnosis, aiming to manage symptoms and enhance life quality from the beginning. The “second transition” arises as the patient’s condition becomes more complex, necessitating specialized PC services for advanced symptom control and intensive psychosocial support. The “third transition” represents the final stage, centering on end-of-life care focusing on comfort, dignity, and emotional and spiritual support, ensuring a peaceful closure for patients and their families [12].

A systematic method could facilitate the early identification of patients with advanced progressive illnesses likely to have unmet PC needs and facilitate end-of-life care planning.

The Surprise Question (SQ) has been proposed as a prompt for the early identification of patients who may benefit from PC. It involves a single reflective query: “Would you be surprised if this patient were to die within the next 6 to 12 months?” Given its simplicity and lack of reliance on complex scoring systems, the SQ has been widely adopted in clinical practice to help healthcare professionals identify patients with potential PC needs. Nevertheless, its subjective nature means that its accuracy may vary depending on both the characteristics of the patient population and the clinician’s judgment. In contrast, the Palliative Care Screening Tool (PCST) offers a more structured approach. It integrates clinical variables—such as functional status and comorbidities—into a scoring algorithm that estimates the patient’s expected survival time. While both the SQ and PCST are established instruments used to support clinical decision-making in the context of PC, comparative data regarding their prognostic performance remain limited. Emerging evidence suggests that using these tools in combination may yield greater predictive accuracy than employing either tool independently.

Identifying patients likely to have unmet PC needs should focus on anticipating their needs and predicting functional decline to create a plan proactively. In this setting, several screening tools have been developed (Table 1). Most tools use either prediction of death or deterioration or both to identify patients. Some tools are applicable in both primary care and hospital settings. Moreover, some utilize the SQ alongside other indicators for a thorough evaluation, while others are tailored to specific patient conditions and do not have a fixed cutoff value. Nevertheless, evaluating these tools is limited because of a lack of a valid comparator, so their actual clinical utility is still being determined [13,14,15]. Further research on effective strategies for identifying patients with potential PC needs would be beneficial.

As the early identification of patients in need of PC could meet patients’ treatment goals and improve their quality of life, it is imperative to screen these patients using a highly accurate screening tool. The NECPAL CCOMS-ICO tool, developed by the Catalan Institute of Oncology in Spain, aims to identify early patients with advanced chronic conditions requiring PC across the healthcare system [16]. This instrument can be used in outpatient and in-hospital environments. It combines a simple SQ, ‘Would you be surprised if this patient died within the next 12 months?’, with six parameters. The parameters of the NECPAL instrument include (1) Palliative Needs Identified, where healthcare professionals determine if the patient requires PC; (2) Functional Decline, assessing the patient’s ability to perform daily activities; (3) Nutritional Decline; (4) Multi-Morbidity, indicating the presence of more than two chronic diseases added to the principal condition; (5) Use of Resources, relating to the frequency of emergency admissions or increased demand for medical interventions within six months; and (6) Specific Disease Criteria, which assesses the severity or progression of the specific chronic conditions. A patient is NECPAL+ if the SQ is answered positively (SQ+) and at least one parameter is met. Its latest version, 4.0, 2021, for the first time, incorporates a patient survival estimation (Supplementary Materials). NECPAL+ patients are categorized into three prognostic stages to estimate survival: Stage I (SQ+ and 1–2 parameters), with an estimated survival of 38 months; Stage II (SQ+ and 3–4 parameters), with 17.2 months; and Stage III (SQ+ and 5–6 parameters), with 3.6 months (Supplementary Material S1) [17]. Incorporating mortality and prognosis assessments into the tool could more accurately pinpoint the optimal time to initiate PC. However, external validation is essential to improve their clinical performance.

## 2. Aims and Objectives

This prospective observational cohort study aims to investigate the accuracy of the NECPAL version 4.0 (2021) tool—developed by the NECesidades PALiativas, a Collaborating Centre of the World Health Organization at the Catalan Institute of Oncology—in identifying patients with advanced chronic conditions who require early PC and analyzes its effectiveness as a mortality predictor.

### 2.1. Primary Objectives

Two primary objectives of the research are considered:

a. To explore, in an accessible population of patients assisted within the Community of Madrid, Spain public health system, the proportion of patients with advanced chronic illnesses who require PC using the NECPAL version 4.0 (2021) tool.
b. To investigate the instrument’s accuracy in predicting mortality in this population.

### 2.2. Secondary Objectives

a. To describe in this population the functional and nutritional deterioration, the prevalent advanced chronic diseases, the demand and need for PC by the patient, their family, and the professional.
b. To compare the perceived quality of life among NECPAL+ patients receiving PC, those not receiving it, and NECPAL- patients using the EuroQol 5D tool (EQ-5D).

## 3. Methods

### 3.1. Study Design

We are conducting a longitudinal, prospective, observational study based on a cohort of non-randomized patients, following the STROBE recommendations for observational studies [18].

The goal is to identify existing screening tools for the identification of patients with advanced progressive diseases who are likely to have PC needs in primary healthcare and evaluate their accuracy.

This study analyzes the predictive capacity of the latest version, 4.0, of the NECPAL instrument for identifying patients with advanced progressive diseases who are likely to have PC needs. Moreover, it evaluates the accuracy of mortality predictions at three time points (3, 17, and 38 months), focusing on specific clinical outcomes and patient-reported measures. The study commenced on 1 February 2024, with an anticipated completion date of 1 May 2030.

The study was approved by the Ethics Committee of the Hospital Universitario Clínico San Carlos (code 22/202–E) and conforms to the Declaration of Helsinki, alongside complying with the Good Clinical Practices (GCP) of the International Conference of Harmonization (ICH). Written informed consent is obtained from all participants, with provisions for adapted communication strategies for patients with limited cognitive capacity whose legally authorized representatives provide consent.

### 3.2. Setting

The study is planned to span six years, including a recruitment period of 30 months followed by a 12-month follow-up period for each patient. Patients will be recruited from both outpatient consultations and hospital admissions.

### 3.3. Participants

Patients with advanced chronic conditions will be invited to take part in the study. Oral and written information will be given.

#### 3.3.1. Inclusion Criteria

Patients are eligible for inclusion in the study if they fulfill the following criteria: adult (at least 18 years of age) with cancer or non-oncological advanced chronic condition. Non-cancer advanced chronic conditions are defined as progressive and incurable diseases with no reasonable possible therapeutic response by the criteria established by the Spanish Society of Palliative Care (SECPAL) [19]. These conditions include target organ damage, such as heart, pulmonary, liver, and kidney diseases; neurodegenerative disorders, dementia, and cerebrovascular disease; and peripheral vascular illnesses (Table 2). Cancer describes neoplasms that cannot be cured [20,21].

#### 3.3.2. Exclusion Criteria

a. Patients who are likely to be transferred to other facilities or expected to move out of the hospital’s geographic area during the study period.
b. Patients with a projected life expectancy of less than 3 months. Patients with an estimated life expectancy of less than three months are excluded from this study, as the NECPAL version 4.0 tool is designed for the early identification of patients with advanced chronic conditions who may benefit from anticipatory palliative care planning. The prognostic stratification provided by the tool begins at a minimum horizon of three months (Stage III), and including patients with a shorter expected survival could impair the assessment of its predictive performance and clinical applicability in earlier stages of disease. This criterion ensures that the study remains focused on the population for whom proactive palliative interventions may still have a meaningful impact.

### 3.4. Patients Recruitment

Investigators will identify potential participants through electronic health records in various settings, including outpatient consultations, hospitalized patients, or follow-up visits. Principal Investigators will complete an eligibility criteria checklist for all considered cases. Clinical information will be used to assess eligibility. To ensure a fair and objective study, patients will be stratified based on age, gender, underlying chronic conditions, and NECPAL tool results (positive or negative) to create balanced groups.

At the time of writing this manuscript, 105 patients were included in the study.

### 3.5. Principal Outcome Measures

- Predictive accuracy of NECPAL version 4.0 (2021) tool for identifying patients who need PC.
- Mortality prediction accuracy at 3, 17, and 38 months.
- Quality of life.

### 3.6. Data Collection

The data for each study participant will be incorporated into the electronic Case Report Form (eCRF) using REDCap [22] designed specifically for this purpose. The data recorded in the eCRF will be directly imported from the computerized medical records.

### 3.7. Data Variables

#### 3.7.1. Primary Dependents Variables

- Positive NECPAL identification.
- Mortality at 3, 17, and 38 months.

#### 3.7.2. Independent Variables

- NECPAL instrument: SQ (+/−) and number of positive parameters.
- The patient receives PC (Yes/No).
- Quality of Life: assessed using the EuroQol 5D-5L questionnaire.
- Demographics: age, gender.
- Chronic conditions: cancer and non-oncological advanced chronic conditions.
- Setting: outpatients and hospitalized patients.
- Functional status: Barthel Index score
- Health variables: primary healthcare center visits, home visits by primary care, hospital emergency visits, number of active specialist consultations, number of daily medications consumed.
- Nutritional variables: albumin, lymphocytes, prealbumin, retinol-binding protein, transferrin, cholesterol, body mass index (BMI), dietary intake record.

## 4. Study Procedures

The study is being conducted at Hospital Universitario Infanta Leonor-Virgen de la Torre, a secondary-level hospital in Madrid, Spain, involving outpatients and inpatients. Eligible participants are adults aged 18 years or older with at least one advanced chronic condition or cancer, identified by physicians through electronic medical records. Advanced chronic conditions are defined, according to the criteria established by SECPAL guidelines (Table 2). Cancer describes neoplasms that cannot be cured. Physicians apply the NECPAL tool to each case, beginning with the SQ, which asks, “Would you be surprised if this patient were to die in the next 12 months?”. If the healthcare professional answers “no” to this question, the patient is considered SQ+. To be considered NECPAL+, an SQ+ patient must also meet at least one parameter from the NECPAL tool. If the professional answers “yes” to the SQ (SQ−), or if an SQ+ patient does not meet any additional NECPAL parameters, the patient is classified as NECPAL−. All patients classified as NECPAL+ are deemed to need PC. It is recorded whether the NECPAL+ patient is receiving PC or not. Mortality status (alive or deceased) is assessed and recorded at 3 (NECPAL stage I), 17 (NECPAL stage II), and 38 months (NECPAL stage III), including the date and cause of death, if applicable. Mortality data are collected through telephone follow-ups and chart reviews. The quality of life of the study population is assessed using the EuroQol-5D-5L instrument (EuroQol Research Foundation, Rotterdam, The Netherlands). The questionnaire is administered at inclusion, 3 (NECPAL stage I), 17 (NECPAL stage II), and 38 months (NECPAL stage III), if applicable. Quality of life is compared among NECPAL+ patients receiving PC, those not receiving it, and NECPAL− patients. No assessments beyond those required for usual care are conducted.

Although the study protocol does not include scheduled re-assessments of NECPAL- patients during follow-up, these individuals will continue to receive routine clinical care within the healthcare system. Any significant changes in their clinical status may prompt re-evaluation by the attending medical team, including the potential re-application of the NECPAL tool if deemed appropriate. While these additional assessments are not captured prospectively in the study database, they reflect the clinical reality and the dynamic nature of patients’ needs over time. This approach ensures that patients who may not initially meet criteria for early palliative care are not excluded from appropriate care if their condition evolves.

## 5. Statistical Analysis

### 5.1. Sample Size Calculation

To determine the appropriate sample size for our study, we considered a population of approximately 350,000 inhabitants. Employing a 95% confidence level (Z = 1.96), a 5% margin of error (E = 0.05), and assuming maximum variability (*p* = 0.5), the initial sample size was calculated to be 384 participants. We applied a 10% oversampling rate to account for potential non-responses, increasing the target sample size to approximately 420 participants.

### 5.2. Analysis

Quantitative variables will be expressed as means with standard deviations, and categorical variables as absolute frequencies and percentages. To evaluate the association between NECPAL+/– and mortality at 3, 17, and 38 months, we will analyze sensitivity, specificity, positive predictive value (PPV), and negative predictive value (NPV). Mortality rates will be compared using the chi-square test for equality of proportions. The Kaplan–Meier method will be used to estimate non-parametric survival curves, and the log-rank test will be applied to assess their differences. Additionally, a semi-parametric Cox proportional hazards regression model will be performed to predict the risk of death. Statistical analyses will be performed using the Statistical Package for Social Science version 29.0 (SPSS Inc., Chicago, IL, USA). A value of *p* < 0.05 will be defined as statistically significant.

## 6. Ethical Considerations

This study has been designed by the Helsinki Declaration and the current Spanish legislation on medical research (Biomedical Research Act 17/2007 of July 3) and data protection (Organic Law 3/2018 of December 5 on the Protection of Personal Data and Guarantee of Digital Rights and the General Data Protection Regulation). It will follow Good Clinical Practice Standards and current legal regulations.

### 6.1. Information and Obtaining Patient Informed Consent

Each patient or their legal representative will be informed orally and in writing through the Patient Information Sheet, detailing the study’s objectives, methods, and duration. The patient or their legal representative will be given sufficient time to read the Patient Information Sheet and will have the opportunity to ask questions. The researcher will obtain written Informed Consent from the patient or their legal representative, including the date and the signatures of both the researcher and the patient/legal representative. An electronic signature (e-consent) option will also be offered. This signing procedure will be optional and will only be carried out if the patient or legal representative consents. Researchers will implement robust security measures for the electronic signature process. Once the signing procedure is completed, the e-consent will be securely stored on the REDCap data collection platform with the same patient ID number in the study, and a copy will be given to the signer. In no case will study procedures be initiated before signature.

### 6.2. Data Confidentiality

The data collected during the study will be kept strictly confidential and accessed only by team members. Researchers must ensure the data’s accuracy, completeness, and legibility; this responsibility is underscored by our strict compliance with the 2018 Data Protection Act. We have put in place regular checks and monitoring to ensure compliance, providing a secure and reassuring data management process. The eCRF meets the requirements for accuracy and reliability. The participants will be assigned an individual-specific number, and their details will be anonymized on the database. At all times, researchers will be responsible for the fidelity and veracity of all recorded data. The study data will be destroyed once their analysis is completed.

## 7. Discussion

Identifying patients likely to have unmet PC needs is crucial to ensuring they receive the appropriate care at the right time. This identification process should not only lead to a referral to specialist PC services but also guarantee a comprehensive and holistic assessment of patients. This comprehensive assessment is central to improving policy-making, service planning, and care delivery, and we must address all aspects of patient care.

The ability of current screening tools to identify patients with advanced progressive diseases who are likely to have PC needs in primary care is limited. In a systematic review, the performance metrics for the screening tools were generally poor [15]. Further research is required to identify standardized screening processes. This research, based on predicting mortality and deterioration, anticipating the PC needs, and predicting the rate and course of functional decline, would prompt a comprehensive assessment. This assessment is not just important but crucial to identify and meet their needs on time.

The identification process of these patients should not be based solely on predicting mortality, but it should also focus on anticipating their needs whenever they occur and predicting the rate and course of functional decline in order to make a proactive PC plan. Our study seeks to explore the potential of the NECPAL version 4.0 (2021) tool in our area of care. The unique feature of NECPAL, which allows for the simultaneous establishment of the appropriateness of PC and the prognosis prediction, could significantly improve patient care. Previous studies have evaluated the instrument in predicting mortality using a short cut-off (12 months), showing a moderate capacity [23,24]. To our knowledge, this study is the first to analyze the predictive capacity of the latest version of the tool that incorporates long-term mortality, offering a more comprehensive understanding of patient needs and providing a solid foundation for future research. The potential impact of our findings could inspire a new wave of research and innovation in the field of PC.

NECPAL is a straightforward instrument based on variables commonly obtained in daily practice, making it easy to incorporate into healthcare systems. The mortality prediction into risk groups could allow classified patients to undergo the three transitions. However, research is needed to evaluate predictive ability and transportability.

## 8. Conclusions

Implementing validated and standardized screening tools would transform the identification process of people with advanced progressive diseases and improve timely access to PC. NECPAL version 4.0 (2021) instrument could help physicians detect those patients in whom PC should be integrated earlier, with the aim of an integral response to the needs of patients suffering from multiple chronic diseases and to plan care.

## Figures and Tables

**Table 1:** Tools for identifying patients with potential palliative care needs.

Tool	Target Patients	Setting	Tool-Positive
**SPICT**	All	Primary care and hospital	Tool-positive criteria: SPICT (2019 version): No formal cut-off; identification is based on clinical judgment informed by the presence of general and/or clinical indicators SPICT-J (Japanese version): Tool-positive if ≥2 general indicators or ≥1 clinical indicator are present SPICT-ES (Spanish version): Tool-positive if ≥2 general indicators and ≥1 clinical indicator are present
**NECPAL CCOMS-ICO**	All	Primary care and hospital	NECPAL: SQ+ and ≥1 parameter
**RADPAC**	COPD, heart failure and cancer patients	Primary care	No cut-off value
**GSF-PIG**	All	Primary care and hospital	GSF PIG: SQ+ and ≥1 general indicator or ≥1 specific indicator
**PALLIA-10**	Hospitalized patients with chronic diseases or cancer	Primary care and hospital	PALLIA-10 score ≥3 indicates the need for palliative care referral
**SQ**	All	Primary care and hospital	SQ+
**The double SQ**	All	Primary care and hospital	The double SQ: A combination of SQ1 ‘no’ and SQ2 ‘yes’
**AnticiPal**	All	Primary care	At least one inclusion criterion is fulfilled, and no exclusion criteria are present. Inclusion criteria: (1) Malignancy codes (e.g., breast cancer), (2) Other single Read Codes (e.g., dementia), (3) Combinations of Read Codes (e.g., chronic kidney impairment and neuromuscular diseases)
**Rainone**	All	Primary care	No formal cutoff; clinical consideration is guided by a “No” response to the SQ and/or affirmative responses to items on clinical complexity, symptom burden, and functional decline.
**TW-PCST**	All	Hospital	ABCD (“A” for advanced aspects of the disease or severe symptoms, “B” for biomarkers or biological indicators, “C” for specific clinical criteria that indicate deterioration, and “D” for the dynamics of deterioration, such as the rate at which the patient’s condition is worsening) score ≥ 2

Abbreviations: SPICT, Supportive and Palliative Care Indicators Tool; NECPAL CCOMS-ICO, Necesidades Paliativas—Centre Collaborateur de l’Organisation Mondiale de la Santé-Institut Català d’Oncologia; RADPAC, RADboud indicators for PAlliative Care needs; GSF PIG, Gold Standards Framework Prognostic Indicator Guidance; PALLIA-10, Palliative care screening tool with 10 indicators; SQ, Surprise Question; AnticiPal, Anticipatory Palliative Care Identification Tool; COPD, Chronic Obstructive Pulmonary Disease; TW-PCST, Taiwan Wu–Palliative Care Screening Tool.

**Table 2:** Criteria for advanced chronic conditions.

Target Organ Damage	Criteria
Heart disease *	Symptoms resistant to conventional treatment, elevated brain natriuretic peptide levels Left ventricular ejection fraction less than 30 percent New York Heart Association Class III or IV Two or more hospitalizations or emergency visits in the past year Angina in elderly patients not eligible for coronary revascularization
Pulmonary disease *	Two or more hospitalizations or emergency visits in the past 12 months due to exacerbations Modified Medical Research Council Dyspnea Scale score of 4 Twenty-four-hour oxygen therapy Forced expiratory volume in one second is less than 30 percent
Liver disease *	Child-Pugh Classification Stage B with more than 7 points Ascites resistant to diuretics Hepatorenal syndrome Grade III-IV hepatic encephalopathy Variceal bleeding is resistant to therapy
Kidney disease *	Creatinine clearance is less than or equal to 30 milliliters per minute, not eligible for dialysis
**Dementia**	Global Deterioration Scale Stage 6 (severe functional impairment) and 7 (mutism added)
**Cerebrovascular disease**	Modified Rankin Scale score of 4 or 5
Neurodegenerative disease *	Amyotrophic Lateral Sclerosis: Barthel Index score less than 40 Multiple Sclerosis: Expanded Disability Status Scale score greater than or equal to 5 Parkinson’s Disease: Hoehn and Yahr Classification Stage 4
Peripheral vascular disease *	Disease not amenable to revascularization Pain in limbs at rest Skin changes, ulcers, or necrosis
Oncological disease *	Cancer spread beyond the original site, including distant metastases or large locally advanced tumors Resistance to specific cancer treatments Severe symptoms: uncontrolled pain, significant weight loss, or extreme fatigue

* The presence of any single criterion is sufficient to define the condition as advanced. The criteria are not cumulative.

## Data Availability

Not applicable, as it is a protocol.

## Supplementary Materials

The following supporting information can be downloaded at https://www.mdpi.com/article/10.3390/mps8030045/s1, S1: NECPAL 4.0 PROGNOSTIC (2021) [25,26,27,28].

## Abbreviations

The following abbreviations are used in this manuscript:

NECPAL CCOMS-ICO	NECesidades PALiativas-Centro Colaborador de la Organización Mundial de la Salud-Institut Català d’Oncologia.
PC	Palliative Care.
SQ	Surprise Question.
