# Supplementary material for: External Validation of the NECPAL CCOMS-ICO Prognostic Tool for Early Palliative Care and Mortality Prediction in Patients with Advanced Chronic Conditions: A Prospective Observational Study Protocol
Source: Methods Protoc. 2025 May 1;8(3):45. doi: 10.3390/mps8030045 (PMC12101327; doi:10.3390/mps8030045)
Supplement: Supplementary file 1 [file mps-08-00045-s001.zip › mps-3463405-supplementary.pdf]

PRACTICAL RECOMMENDATIONS FOR IDENTIFYING AND ESTABLISHING PROGNOSTIC APPROACH OF PEOPLE WITH ADVANCED CHRONIC CONDITIONS AND PALLIATIVE CARE NEEDS IN HEALTH AND SOCIAL SERVICES

NECPAL 4.0 PROGNOSTIC (2021)

Authors and researchers: Xavier Gómez-Batiste, Jordi Amblàs, Pamela Turrillas, Cristian Tebé, Judit Peñafiel, Agnès Calsina, Xavier Costa, Josep Maria Vilaseca, Rosa Maria Montoliu

Collaboration team: Sarah Mir, Elba Beas, Marina Geli

CC BY NC

Càtedra
de Cures Pal·liatives
UVIC·UCC
ICO - INSTITUT CATALÀ D'ONCOLOGIA

With the support of:

Generalitat de Catalunya
Programa de prevenció i atenció a la cronicitat

Generalitat de Catalunya
Pla interdepartamental d'atenció i interacció social i sanitària

INTRODUCTION AND BACKGROUND: THE NECPAL PROGRAM

The NECPAL Program was built to respond the challenge of timely, comprehensive, and integrated palliative approach and care for people with advanced chronic conditions from all causes in all settings of the health ad social systems, and has included research, education, and knowledge transfer. The tool has been translated and validated in many cultures, and used intensively in many countries.

The NECPAL utilities

The NECPAL utilities have been so far the screening of people with palliative care needs in services, which allows the determination of their prevalence, and to elaborate a basic checklist of their needs. In the evolutive updating of the tool, it has been improved, adding some criteria as the geriatric and psychosocial issues, and defining more precisely the existing parameters.

Developing the NECPAL 4.0 PROGNOSTIC

The aim of designing the NECPAL 4.0 PROGNOSTIC is to explore its prognostic utility, and has included different steps: literature review, expert's consensus, building a predictive model, and testing it's validity with a retrospective analysis of a cohort recruited previously.

HOW TO USE IT IN PRACTICE IN HEALTH AND SOCIAL SERVICES?

Steps: the first steps are similar to the previous versions:

1. Review the list of people attended by the service.
2. Elaborate a list of those persons with chronic conditions specially affected.
3. Apply the surprise question to doctors and nurses about well-known patients: “Would you be surprised inf this patient die in one year?” with clinical criteria.
4. In those patients in which the response was “I’m or we will not be surprised”, explore the different NECPAL generic parameters and the specifics for conditions.

The result of this procedure will be **list of patients having palliative care needs and a limited life prognosis** *(Figure 1: NECPAL «classic» 3.1)*

Figure 1. NECPAL 3.1 «classic» with all components

Surprise question (to/among professionals):
Would you be surprised if this patient dies within the next year?

▶ **YES,** I would be surprised → **NOT NECPAL**

▶ **NO,** I would not be surprised

**"Demand" or "Need"**	- Demand: Have the patient, the family or the team requested in implicit or explicit manner, palliative care or limitation of therapeutic effort?
	- Need: identified by healthcare professionals from the team
**General Clinical Indicators: 6 months**<br>- Last 6 months<br>- Not related to recent/reversible intercurrent process	- Nutritional Decline
	- Functional Decline
	- Cognitive Decline
**Severe Dependence**	- Karnofsky <50 o Barthel <20
**Geriatric Syndromes**	- Falls - Pressure Ulcers<br>- Dysphagia - Delirium<br>- Recurrent infections
**Persistent symptoms**	Pain, weakness, anorexia, digestive...
**Psychosocial aspects**	Distress and/or Severe adaptive disorder
	Severe Social Vulnerability
**Multi-morbidity**	>2 chronic diseases (from the list of specific indicators)
**Use of resources**	Evaluate Demand or intensity of interventions
**Specific indicators of illness severity/progression**	Cancer, COPD, CHD, Liver, Renal, CVA, Dementia, Neurodegenerative diseases, AIDS, other advanced illnesses

If there is at least 1 NECPAL Parameter: **NECPAL +**

NECPAL TOOL VERSION 4.0 2021

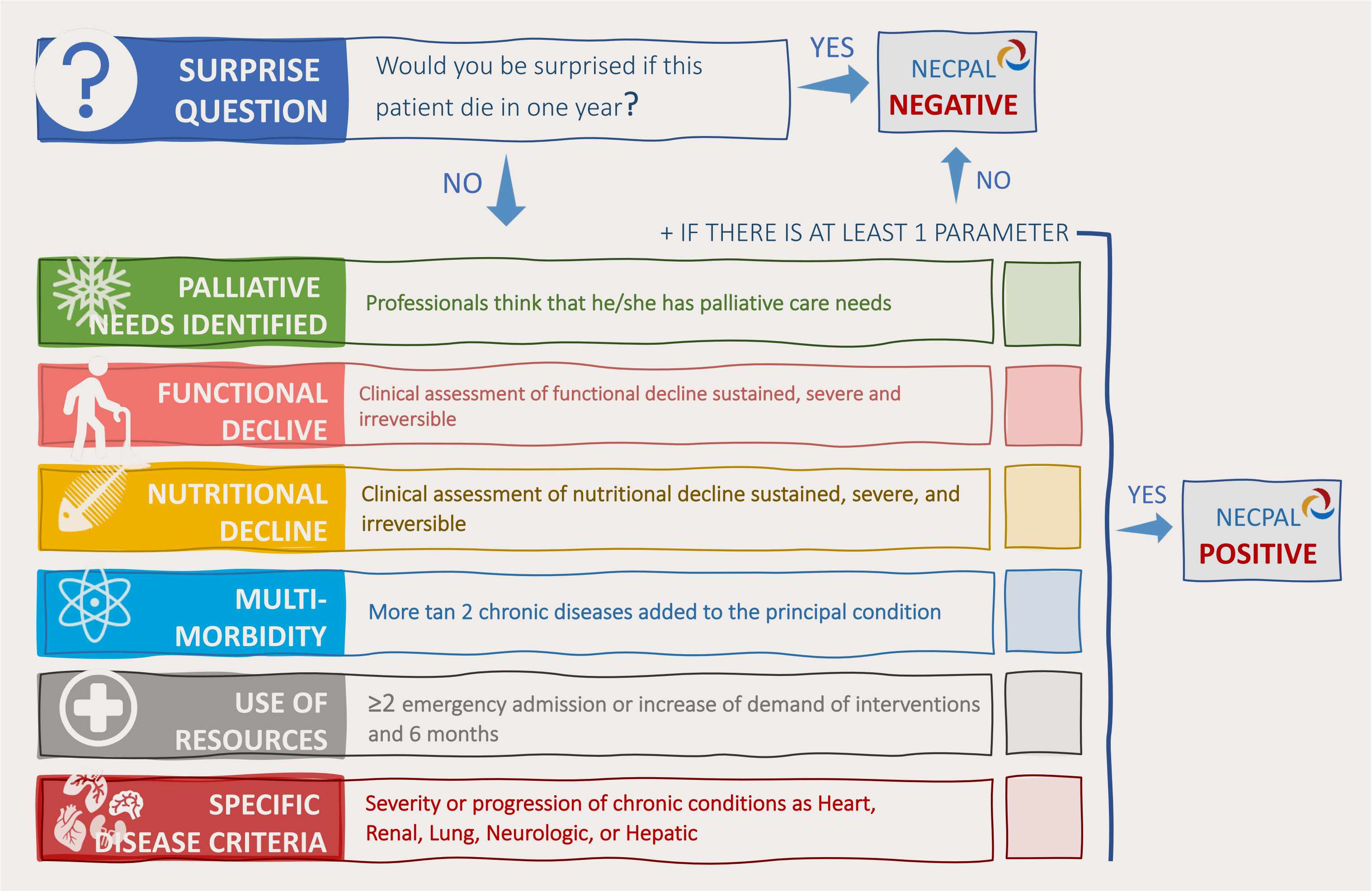

> Need's Checklist: Identification of palliative care needs to insert a palliative approach

1. Realize a rapid checklist of the need's dimensions.
2. Complement with additional indicators and parameters if needed.
3. Elaborate aims and actions to respond to the identified needs.
4. Elaborate a comprehensive therapeutic plan.

The result of this procedure permits to identify palliative care needs and elaborate a comprehensive therapeutic plan:

Actions for the comprehensive care of people identified

1. Multidimensional assessment
2. Assessment of the stage of diseases and conditions and possible evolution
3. Identify values, preferences, and start advance care planning
4. Identify and care principal career
5. Identify and actívate referent profesional
6. Multidimensional Therapeutic Plan
7. Case management and integrated care with other services

> "Situational" Checklist: identification of the prognostic risk to elaborate the prognostic approach:

- Situational prognostic checklist
- Risk estimation
- Criteria for prognostic approach

Listing the parameters with prognostic utility (palliative needs identified by professionals, functional decline, nutritional decline, multimorbidity, increased use of resources, and parameters of the specific disease.

The result of this procedure includes the patient MACA in one of these three prognostic stages:

The evolutive stage: can be determined according to the number of parameters affected. If 1-2 or 3-4 or 5-6

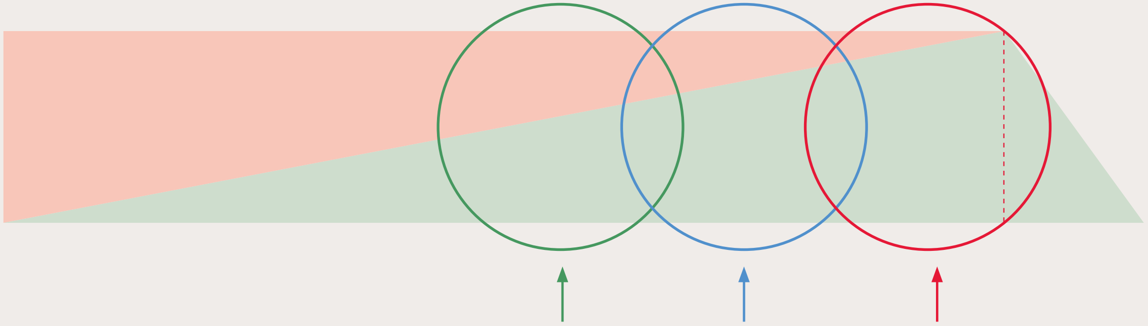

PS
Surprise question

List of Prognostic parameters

- Palliative needs identified by professionals
- Functional decline
- Nutritional decline
- Multimorbidity
- Increase in use of resources
- Disease-Specific parameters

Stage I

- PS + 1-2 parameters
- Median: 38 months

Stage II

- PS + 3-4 parameters
- Median: 17.2 months

Stage III

- PS + 5-6 parameters
- Median: 3.6 months

ASPECTS TO CONSIDER

How to manage the prognostic assessment in clinical practice

1. The prognosis is one of the elements to consider, added to the needs and demands .
2. The prognostic risk is applied to populations that accomplish criteria, but must be applied with caution to individual patients.
3. Once established, we will have a prognostic situational perspective, which can be valuable for a therapeutic approach.
4. It is recommended to update it regularly.

Risks and benefits of the prognostic approach

1. The most relevant benefit of the prognostic assessment is to contribute to the situational assessment and permits redefinition of the therapeutic aims, introducing gradually a palliative approach.
2. This assessment must be shared with patients, relatives and team, with the rithm, intensity, and concretion adapted individually to the adjustment and preferences of patients.
3. The most relevant risk consists in the automatic individual application of a population-based risk.
4. There are other identified risks, as those related to loosing curative opportunities, stigmatization, impact on patients and families, and must be prevented.

REFERENCES AND SOURCES

References:

Amblàs-Novellas J, Murray SA, Espaulella J, et al. Identifying patients with advanced chronic conditions for a progressive palliative care approach: a cross-sectional study of indicators related to end-of-life trajectories. BMJ Open 2016;6:e012340.

Boyd K, Murray SSA. Recognising and managing key transitions in end of life care. BMJ 2010;341:c4863.

Gómez-Batiste X, Blay C, Broggi MA, et al. Ethical Challenges of Early Identification of Advanced Chronic Patients in Need of Palliative Care. J Palliat Care 2018;082585971878893.

Gomez-Batiste X, Martinez-Munoz M, Blay C, et al. Identifying patients with chronic conditions in need of palliative care in the general population: development of the NECPAL tool and preliminary prevalence rates in Catalonia. BMJ Support Palliat Care [Internet] 2013;3(3):300–8. Available from: http://spcare.bmj.com/content/early/2012/12/14/bmjspcare-2012-000211

Gómez-Batiste X, Martínez-Muñoz M, Blay C, et al. Utility of the NECPAL CCOMS-ICO© tool and the Surprise Question as screening tools for early palliative care and to predict mortality in patients with advanced chronic conditions: A cohort study. Palliat Med 2017;31(8):754–63.

Gómez-Batiste X, Turrillas P, Tebé C, Calsina-Berna A, Amblàs-Novellas J. NECPAL tool prognostication in advanced chronic illness: A rapid review and expert consensus. BMJ Support Palliat Care 2020;1–11.

Thoonsen B, Vissers K, Verhagen S, et al. Training general practitioners in early identification and anticipatory palliative care planning: a randomized controlled trial. BMC Fam Pract [Internet] 2015;16:126. Available from: http://www.biomedcentral.com/1471-2296/16/126

Turrillas P, Peñafiel J, Tebé C, Amblàs J and Gómez-Batiste X. NECPAL prognostic tool: a palliative medicine retrospective cohort study. BMJ Supportive & Palliative Care Epub ahead of print: 16 February 2021. doi:10.1136/bmjspcare-2020-002567

Sources:

- www.catedrapaliativos.com
- www.iconcologia.net
- https://salutweb.gencat.cat/ca/ambits_actuacio/linies_dactuacio/estrategies_salut/cronicitat/documentacio-pla-de-salut-2016-2020/
- Training activities information available at: https://www.catedrapaliativos.com/formacio/
